# Barriers to participation in HIV vaccine trials and cancer trials: a cost-gain analysis

**DOI:** 10.1186/1742-4690-9-S2-P109

**Published:** 2012-09-13

**Authors:** S Dhalla, G Poole

**Affiliations:** 1 University of British Columbia, Vancouver, Canada; 2 School of Population and Public Health, University of British Columbia, Vancouver, Canada

## Background

Previous studies examining barriers and willingness to participate in HIV vaccine trials have demonstrated the role of factors identified by the Health Belief Model. Barriers to cancer trials can also be understood in terms of a theoretical framework consisting of the locus of the barrier (personal vs. social) and the nature of the barrier (risk vs. cost). In this systematic review of review articles, we extend this framework to another life-threatening disease, cancer. The purpose is to improve conceptual clarity about volunteering in clinical trials by comparing and contrasting barriers in these two areas.

## Methods

In 2012, two people independently searched the Cochrane Database for Systematic Reviews, Pubmed, Embase, and Google Scholar to identify review articles examining cancer trial barriers to participation. Search terms used were: “cancer”, “oncology”, “cancer trials”, “oncology trials”, “clinical trials”, “medical research”, “willingness to participate”, “barriers”. Review articles were also retrieved from our search examining motivators to participation in cancer research and from bibliographic references.

## Results

We retrieved 19 review articles from 2000-2012 examining barriers to participation in cancer trials. “Reduced quality of life” / “distrust of institutions” / “loss of control” were personal risks (PR). “Perceptions of the provider” / “subjective norms” were social risks (SR). “Side effects” / “experimental nature of the trial” were personal costs (PC). Misconceptions included “confidentiality concerns”. Consistent with HIV vaccine trials, most barriers with regards to cancer trials were related to PR and PC. More misconceptions were identified in HIV vaccine preparedness studies (VPS).

## Conclusion

Personal risk, PC, and SR barriers were similar to those identified in HIV VPS, but more misconceptions were identified in the latter. Understanding barriers can result in better recommendations on how to overcome these barriers. A limitation is that cancer populations are different than those affected by HIV.

